# Chaperone-Mediated Autophagy in Neurodegenerative Diseases: Molecular Mechanisms and Pharmacological Opportunities

**DOI:** 10.3390/cells11142250

**Published:** 2022-07-20

**Authors:** Yi-Ting Wang, Jia-Hong Lu

**Affiliations:** State Key Laboratory of Quality Research in Chinese Medicine, Institute of Chinese Medical Sciences, University of Macau, Macau SAR 999078, China; mc15359@um.edu.mo

**Keywords:** chaperone-mediated autophagy, autophagy, LAMP2A, HSC70, α-synuclein, neurodegenerative disease, Parkinson’s disease, Alzheimer’s disease, Huntington’s disease, small molecule

## Abstract

Chaperone-mediated autophagy (CMA) is a protein degradation mechanism through lysosomes. By targeting the KFERQ motif of the substrate, CMA is responsible for the degradation of about 30% of cytosolic proteins, including a series of proteins associated with neurodegenerative diseases (NDs). The fact that decreased activity of CMA is observed in NDs, and ND-associated mutant proteins, including alpha-synuclein and Tau, directly impair CMA activity reveals a possible vicious cycle of CMA impairment and pathogenic protein accumulation in ND development. Given the intrinsic connection between CMA dysfunction and ND, enhancement of CMA has been regarded as a strategy to counteract ND. Indeed, genetic and pharmacological approaches to modulate CMA have been shown to promote the degradation of ND-associated proteins and alleviate ND phenotypes in multiple ND models. This review summarizes the current knowledge on the mechanism of CMA with a focus on its relationship with NDs and discusses the therapeutic potential of CMA modulation for ND.

## 1. Introduction

Neurodegenerative diseases (NDs), one of the major health threats to human, affect 50 million people worldwide [1]. As ND is an age-related disease, population aging also makes ND an urgent health issue. The most common NDs include Alzheimer’s disease (AD), Parkinson’s disease (PD), Huntington’s disease (HD), amyotrophic lateral sclerosis (ALS), frontotemporal lobar degeneration (FTLD), and the spinocerebellar ataxias (SCA) [2]. The common feature of ND is the intracytoplasmic deposition of aggregate-prone proteins in neurons. The removal of undesired, damaged, misfolded, and aggregated proteins is a therapeutic focus of ND.

Autophagy is a self-degradation pathway via lysosome. It plays a homeostatic function and is involved in the pathophysiological process of anti-aging, anti-microbial, anti-tumor, differentiation, development, and immunity [3].

Autophagy is classified as macroautophagy, microautophagy, and chaperone-mediated autophagy (CMA) based on how cargo is delivered to the lysosome. Macroautophagy is the best-studied type of autophagy. During macroautophagy, the cargo is sequestrated by double-membrane vesicles-autophagosomes and transported to the lysosome. In microautophagy, the lysosomes directly uptake the cytosolic compounds through membrane enwrapping. In CMA, heat shock cognate 71 kDa protein (HSC70) chaperones bind to damaged or defective proteins containing KFERQ-like sequences and transport them to the lysosomes via lysosome-associated membrane protein 2A (LAMP2A) [4]. With increasing age, the efficiency of CMA is lowered, which increases the risk of harmful proteins accumulating into insoluble clumps that damage cells. The common feature of AD and other NDs is the presence of toxic protein aggregates in the patient’s brain [5]. Moreover, the large number of defective proteins overwhelm CMA and eventually paralyze it.

This review summarizes current knowledge on the molecular mechanism, the physiological function of CMA, and its relationship with NDs. We also discuss the therapeutic potential of modulation of CMA for ND treatment and list the small-molecule CMA modulators with neuroprotective properties on ND models.

## 2. CMA as a Therapeutic Target for Neurodegenerative Diseases

### 2.1. Molecular Mechanism of CMA

#### 2.1.1. CMA Substrate Recognition

The CMA-targeting motif is a pentapeptide sequence related to KFERQ biochemically, which must contain up to two positively charged residues: arginine (R), lysine (K); up to two of the hydrophobic residues: isoleucine (I), phenylalanine (F), leucine (L), or valine (V); one single negative residue: glutamate (E) or aspartate (D); and one single glutamine (Q) flanked at either the N- or C-terminus of the pentapeptide [6]. This canonical motif can be found in about 40% of the mammalian proteome [7,8,9]. However, the CMA substrate must have the essential KFERQ motif characteristics so that the substrate can be recognized by HSC70, a constitutively expressed molecular chaperone [6].

#### 2.1.2. Transportation of CMA Substrate by HSC70

Once the KFERQ sequence in the CMA substrate binds to HSC70, the substrate is delivered to the lysosomal membrane. The HSC70 belongs to the heat shock protein family, one of the largest groups of chaperones. The HSC70 can be found in the cellular membrane, extracellular exosomes, the nucleus, and the cytosol [10]. Unlike the HSC70 in the cytosol, the lysosomal HSC70 (lys-HSC70) is involved in CMA substrate uptake [11]. When the pathway is triggered by starvation or oxidative stress, the lys-HSC70 amount and the number of lysosomes that contain lys-HSC70 are increased [12]. Cochaperones, including the carboxyl terminus of HSC70-interacting protein, heat shock protein 40 kDa (HSP40), HSP70-interacting protein, and HSP70-HSP90 organizing protein, are also involved in substrate unfolding and lysosomal translocation [4]. In addition, HSP90 can promote stable binding between protein and LAMP2A at the lysosomal membrane [13].

#### 2.1.3. Translocation of CMA Substrate by LAMP2A

LAMP2A contains a cytosolic tail different from the other variants of the LAMP2 genes [14]. The binding between substrate and LAMP2A promotes LAMP2A multimerization to form a translocation complex during CMA. After the substrate translocates into the lysosome lumen, the LAMP2A multimer disassembles into monomers and returns to the cytosol for reuse [15]. The efficiency of the CMA pathway can be affected by the velocity of assembly and disassembly of the LAMP2A translocation complex. Glial fibrillary acidic protein (GFAP) and elongation factor 1 alpha (EF1α) are identified as the regulators of LAMP2A oligomerization. GFAP contributes to the stability of the translocation complex, whereas the EF1α-GTP binding promotes the self-association between GFAP molecules, further disrupting the translocation complex’s stability. Thus, GTP has been shown to be a CMA inhibitor [16]. Moreover, prolonged starvation, oxidative stress, or inhibition of the proteolytic pathway can raise the LAMP2A levels and LAMP2A-positive lysosomes and further increase CMA activity [17].

### 2.2. Physiological Function of CMA

As a recycling and protein quality control mechanism, CMA plays an important role in maintaining cellular homeostasis and exerting specific physiological functions in regulating the cell cycle, cell survival, cell stemness, transcriptional regulation, metabolic pathways, and immune responses [18]. 

#### 2.2.1. Starvation

CMA can be activated during nutritional starvation to promote amino acid recycling for maintaining protein synthesis and gluconeogenesis [18]. After removal of serum from the medium for 8 to 10 h, CMA will be gradually activated and persists at a high level for 3 days while the activation of macroautophagy is shorter [19,20,21]. The starvation-activated CMA can degrade less critical proteins and keep protein synthesis by recycling amino acids [22]. However, the starvation-activated CMA is tissue and cell-type selective, which can be more effectively stimulated in the liver, spleen, kidney, and heart [23]. 

#### 2.2.2. Protein Quality Control

The protein quality control is another most characterized function of CMA, which can be activated by oxidative stress and hypoxic stress [12,24], and during aging and neurodegenerative disease [25]. CMA can electively remove the damaged and misfolded proteins from the cytosol. The upregulation of LAMP2A transcription is involved in the oxidation-induced activation of CMA [26]. Issa et al.’s research on the *Drosophila* brain indicated that oxidative stress increased LAMP2A expression and CMA activation. The expression of LAMP2A further prevented the accumulation of Ref(2)P, the *Drosophila melanogaster* homolog of mammalian SQSTM1/p62, under acute oxidative stress. These results indicate that CMA increases autophagic flux in the *Drosophila* brain and has neuroprotective properties [27]. The hypoxic stress can also effectively activate the CMA process, which reduces damaged protein and promotes neuron survival [24]. 

#### 2.2.3. Metabolic Regulation

Since CMA’s discovery, it has been associated with cellular energetics and activated by starvation. The alterations in carbohydrate and lipid metabolism caused by the selective degradation of the main enzymes by CMA are responsible for cellular energetics, which is also associated with brain aging and ND [28,29,30]. Most glycolytic enzymes contain KFERQ motifs. For example, glyceraldehyde 3-phosphate dehydrogenase (GAPDH) and pyruvate kinase were first identified as CMA substrates [31]. CMA modulation of carbohydrate metabolism has been established in a mouse model. CMA modulates hepatic glycolysis by regulating the levels of glycolytic enzymes and enzymes involved in the tricarboxylic acid cycle process [32]. The aging-related decline in CMA may raise rates of glycolysis and break the energy balance in old organisms [30,32]. In addition, CMA regulates the level of lipid uptake and lipogenesis. CMA promotes lipid metabolism by the two most common mechanisms: lipogenic enzyme degradation and selective deletion of lipid droplet proteins [28]. Some lipid metabolism-related proteins have been identified as CMA substrates, such as lipogenesis enzymes, lipid carriers, and lipid droplet coat proteins [32,33]. Perilipin 2 (PLIN2) and perilipin 3 (PLIN3), lipid droplet coat proteins, are CMA substrates that can mediate the level of triglycerides and the lipid droplets in lipolysis and lipophagy [33]. CMA degradation of PLIN2 and PLIN3 suggested a function for CMA in lipid homeostasis maintenance. Moreover, the lipid accumulation in the cells of macrophage-specific LAMP2A-deficient mice is associated with brain homeostasis and disease [34].

#### 2.2.4. Cell Cycle Control

CMA modulates several cellular mechanisms by regulating protein degradation and controlling the abundance of proteins. Some cell cycle modulators that carry the KFERQ-like motif are involved in the CMA-induced cell cycle process [35]. For example, the level of myocyte enhancer factor-2 (MEF2) protein can regulate neuronal proliferation and survival [36] while CMA mediates the MEF2D degradation. Both wild-type α-synuclein and a PD-associated mutant blocked MEF2D-HSC70 binding, leading to MEF2D accumulation and neuronal death and further increasing the risk of PD [37,38]. MEF2A, another isoform of MEF2, can be degraded under the oxidative stress-induced lysosome destabilization condition [39]. CMA impairment is related to cellular senescence by triggering the DNA damage response, SA-β-gal activity, upregulation of p21, and accumulation of p16 and lipofuscin. Evidence showed that the dysfunctional CMA led to physiological aging and neurodegeneration by accumulating senescent cells [40]. In addition, CMA is associated with ferroptosis. Ferroptosis plays an important role in multiple neurologic diseases such as stroke, PD, and HD. Glutathione-dependent antioxidant enzyme glutathione peroxidase 4 (GPX4), one of the critical regulators of ferroptosis, is degraded via CMA. Furthermore, suppression of CMA stabilized GPX4 to inhibit ferroptosis [41,42].

#### 2.2.5. Immune Responses

Downregulating LAMP2A or HSC70 in the immune system can limit the distribution of cytoplasmic epitopes by class II molecules in antigen-presenting cells [43]. CMA has also exhibited the capacity to help regulate the CD4+ T cell response, as it selectively degrades the ubiquitin ligase Itch and the calcineurin inhibitor regulator of calcineurin 1 (RCAN1), which are two T cell receptor signaling negative regulators. The relationship between an age-dependent decrease in CMA activity in T cells and the impaired T cell function associated with aging has been verified [44]. Moreover, CMA plays an important role in the immunosuppressive function of mesenchymal stromal cells by IFN-γ plus TNF-α-induced activation of NF-κB and STAT1 [45]. 

### 2.3. Role of CMA in Neurodegenerative Diseases

**Figure 1:**
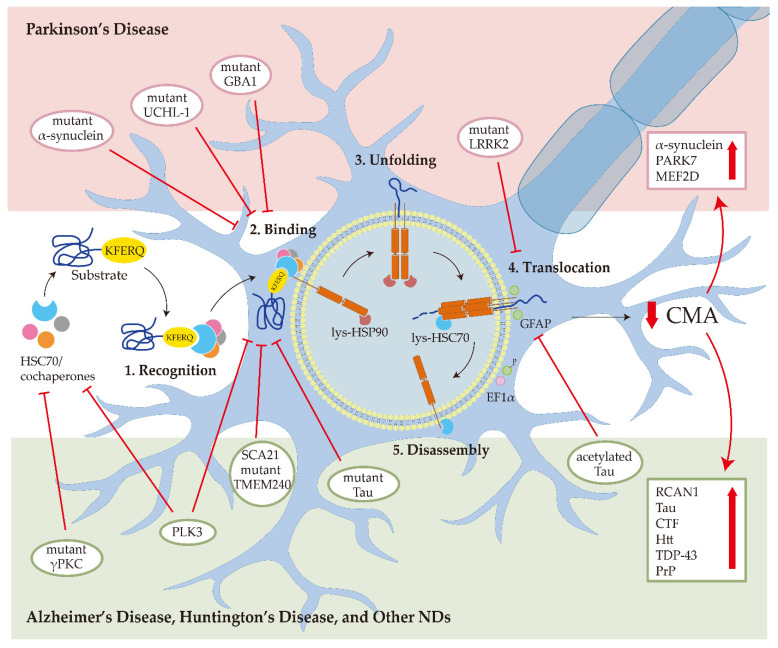
Chaperone-mediated autophagy (CMA) in neurodegenerative diseases (NDs). In Parkinson’s disease, the mutant α-synuclein interacts with LAMP2A with higher affinity and blocks CMA degradation of other substrates; the mutant UCHL-1 and GBA1 reduce CMA activity by aberrantly interacting with the HSC70 and LAMP2A; mutant LRRK2 impairs the formation of translocation complex; the downregulation of CMA can increase the α-synuclein, PARK7, and MEF2D accumulation. In Alzheimer’s disease, mutant Tau binds with LAMP2A and inhibits CMA activity; acetylated Tau prevents the translocation of substrates into the lysosome; the downregulation of CMA can increase the RCAN1, Tau, and CTF levels. In Huntington’s disease, CMA malfunction contributes to Htt aggregation. In prion disease, PLK3 affects the levels of HSC70 and LAMP2A to mediate PrP degradation through CMA. For amyotrophic lateral sclerosis and frontotemporal lobar degeneration, TDP-43 is degraded by CMA. For spinocerebellar ataxias, mutant γPKC interacts with HSC70 and SCA21 mutant TMEM240 blocks the LAMP2A transport.

In the central nervous system (CNS), the hallmarks of many NDs are the misfolding, aggregation, and accumulation of proteins, which result in cellular dysfunction, synaptic damage, and brain damage [46]. Some ND-related proteins are identified as CMA substrate proteins, such as α-synuclein (Figure 1). Several pieces of research showed that CMA activity was reduced in several NDs, suggesting that dysfunctional CMA is implicated in ND [4].

#### 2.3.1. Parkinson’s Disease

PD is the second most prevalent ND and is presently incurable. The clinical symptoms of PD include motor abnormalities such as the symptomatic triad of bradykinesia, resting tremors, and rigidity, and non-motor symptoms such as neurobehavioral disorders, autonomic dysfunctions, sensory impairments, and sleep disturbances [47]. The pathogenic feature of PD is the loss of nigrostriatal dopaminergic innervation. The main pathogenic molecular mechanisms include α-synuclein misfolding and aggregate formation, mitochondrial dysfunction, protein clearance impairment associated with the ubiquitin-proteasome system (UPS) and autophagy-lysosomal, neuroinflammation, and oxidative stress [48], most of which are related to autophagy. α-synuclein (*SNCA*), *Parkin*, ubiquitin C-terminal hydrolase L1 (*UCHL-1*), PTEN-induced kinase 1 (*PINK1*), PARK7 (*DJ-1*), leucine-rich repeat kinase 2 (*LRRK2*), ATPase cation transporting 13A2 (*ATP13A2*), glucocerebrosidase (*GBA*), vacuolar protein sorting ortholog 35 (*VPS35*), Eukaryotic Translation Initiation Factor 4 Gamma 1 (*EIF4G1*), and *PARK16* are identified as the causative genes of PD [49]. Some of these genes have been found as CMA substrates and CMA regulators in PD [50].

Since α-synuclein was identified as a CMA substrate and wild-type α-synuclein was degraded through CMA, PD is the first ND associated with CMA [26]. In CMA, the α-synuclein directly interacts with the key protein player of CMA, LAMP2A [51]. For HSC70, miR-320a (HSC70 miRNA) inhibits CMA and promotes α-synuclein accumulation [52]. Reduced CMA in PD is caused by the loss of LAMP2A and HSC70 proteins, which occurs primarily in brain areas, and also caused by accumulating membrane-associated α-synuclein and other recognized CMA substrates [50]. CMA degrades α-synuclein, whereas pathogenic α-synuclein impairs CMA progress. A30P and A53T are two mutant variants of α-synuclein found in familial forms of PD. These two mutant types of α-synuclein have a higher affinity for the LAMP2A than other substrates. However, they block the degradation of themselves and other substrates through CMA [26]. Similar to the A30P and A53T mutant α-synuclein, dopamine-modified α-synuclein also inhibits its own degradation and other substrates’ degradation via CMA [53]. At the early stage of PD, the reduction of LAMP2A is affected by the increased α-synuclein even before α-synuclein accumulation [54]. These findings suggest that CMA dysfunction is an early event in PD, and increasing the LAMP2A level to promote α-synuclein degradation in CMA is a potential treatment for PD. 

Pathogenic mutations of the LRRK2 are the most common factor for familial PD. LRRK2 has been linked to several putative PD pathogenic mechanisms, including α-synuclein accumulation, Tau hyperphosphorylation, the inflammatory response, oxidative stress, mitochondrial dysfunction, synaptic dysfunction, and autophagy-lysosomal system impairment [55]. A recent study suggested that DNL201, an LRRK2 kinase inhibitor, rescues lysosomal dysfunction in PD patients [56]. CMA could induce LRRK2 degradation in lysosomes. However, the G2019S, a mutant form of *LRRK2*, can suppress CMA via interfering with the formation of translocation complex at the lysosomal membrane [57]. In neurons from *LRRK2* G2019S mice, the mutant *LRRK2* interferes with CMA activity, increases colocalization between LAMP2A and α-synuclein, and causes α-synuclein aggregation [58]. 

In addition to α-synuclein, LRRK2, and their mutants, the UCHL-1, PARK7, MEF2D, VPS35 mutant, and GBA1 mutant are PD-related proteins associated with CMA pathways as well. UCHL-1 and GBA1 mutants aberrantly interact with the HSC70 and LAMP2A, further inducing α-synuclein accumulation by blocking CMA activity [59,60]. PARK7 and MEF2D protein levels can be regulated by CMA activity. CMA was demonstrated to selectively degrade the oxidized and altered PARK7, protecting mitochondria from damaging and affecting cell survival [61]. The mislocalized and inactive MEF2D accumulation caused by CMA dysfunctions is also a potential reason for PD [37]. Pathogenic VPS35 affects LAMP2A retrieval from the endosome to the Golgi complex and increases LAMP2A degradation [62].

There are also some pieces of evidence on the therapeutic role of CMA in the in vivo PD models. In rats, blocking LAMP2A in the substantia nigra caused neurodegeneration. In PD patients’ brains, the level of LAMP2A decreased [63], and the LAMP2A and HSC70 transcripts reduced as a result of an increase in six miRNAs, which include hsa-miR-21*, hsa-miR-224, and hsa-miR-373* targeting *lamp2a*, and hsa-miR-26b, hsa-miR-106a*, and hsa-miR-301b targeting *hsc70* [64]. In leukocytes from PD patients, the LAMP2A and HSC70 mRNA, and LAMP2A and HSC70 protein levels decreased [65,66]. Based on the relationship between CMA activity and PD, activation of CMA is a promising therapeutic strategy for PD. 

#### 2.3.2. Alzheimer’s Disease

AD is the most prevalent form of dementia among senior citizens, and its clinical symptoms are progressive memory impairment and cognitive function loss [67]. The primary lesions of AD are the deposition of extracellular amyloid β (Aβ) and intraneuronal Tau neurofibrillary tangles in particular brain areas [68]. Autophagy has a significant impact on the metabolism of Aβ and Tau proteins. The accumulation of toxic proteins in the AD brain is thought to be caused by autophagy malfunction [69]. Like PD-related proteins, several AD-related proteins have also been identified as CMA substrates. The presence of two CMA-targeting motifs in Tau’s C-terminal region suggests that CMA can degrade the Tau aggregation [70]. Mutant Tau protein can lower CMA activity by interacting with LAMP2A and prevent translocation to the lysosome lumen [70]. The increased level of acetylated Tau affects the translocation of substrates in the lysosome during CMA rather than affecting the binding of substrates to the lysosomal surface. Acetylated Tau reduces the efficiency of its lysosomal translocation due to the decrease in the pH sensitivity for HSC70-Tau binding [71]. RCAN1, another AD-related CMA substrate, can inhibit calcineurin-dependent dephosphorylation of Tau protein. In AD patients, the level of RCAN1 is increased. Activating CMA can promote RCAN1 degradation and may reduce the formation of Tau aggregates [72].

Amyloid precursor protein (APP), a producer of Aβ, contains a CMA motif in the C terminus [73]. Removal of this motif enhanced C-terminal fragment (CTF) levels and sAPP α/β secretion, indicating that this region controls APP processing [73]. In a Tg mice model, CMA reduction did not induce full-length APP accumulation; however, it did dramatically increase the levels of CTFs [74]. Previous research indicated that APP-CTFs promote neurotoxicity and Tau phosphorylation, further increasing the AD risks [75].

#### 2.3.3. Huntington’s Disease and Other NDs

HD, a dominantly inherited late-onset ND, is caused by accumulation and aggregation of mutant huntingtin protein (Htt). Htt is identified as a CMA substrate and interacts with HSC70 and LAMP2A. There is an expended N-terminal polyglutamine (polyQ) tract in Htt [76]. The polyQ-binding protein forces mutant Htt to CMA machinery, resulting in Htt degradation. Interestingly, CMA activity is upregulated in the early stages of HD due to compensatory adjustment of the decreased macroautophagy in HD. For the late stage of HD, the lower level of LAMP2A suggests that CMA is impaired [77].

Other NDs such as prion diseases, ALS, FTLD, and SCA are related to CMA. The prion protein (PrP) is the main content of the prion. Overexpression of polo-like kinase 3 (PLK3) induces the degradation of mutated PrP. In both in vivo and in vitro abnormal PrP models, the levels of HSC70 and LAMP2A are downregulated. In addition, the overexpression of PLK3 can upregulate HSC70 and LAMP2A levels. Taken together, PLK3 mediates the degradation of PrP through CMA [78,79]. Accumulation of transactivation response DNA-binding protein 43 kDa (TDP-43) is the hallmark of ALS and FLTD. CMA can maintain the physiological and pathology forms of TDP-43 as the KFERQ motif in TDP-43 can bind with HSC70 [80]. SCA is a heterogeneous group of progressive ND. SCA14 is caused by mutant γPKC, which is a CMA substrate. The interaction between mutant γPKC and HSC70 blocked CMA activity in neuronal cells [81]. A mutation in transmembrane protein 240 (TMEM240) has been identified to be causative for SCA21, which is another kind of SCA. SCA21-causative TMEM240 mutant blocked the transport function of LAMP2A to reduce CMA activity, and further caused CMA substrate accumulation [82]. The LAMP2A knock-down in cerebellar neurons in the mouse brain resulted in motor impairment, and loss of cerebellar Purkinje cells and interneurons, which indicated that CMA impairment in cerebellar neurons is associated with SCA pathogenesis [83]. 

## 3. Small-Molecule CMA Modulators with Therapeutic Potential in ND Models

The pharmacological enhancement of CMA provides a therapeutic strategy for ND by modulating CMA activity to improve neuronal survival in CNS. Indeed, a series of CMA enhancements have been reported in the last few years. The mechanisms and pharmacological effects of these CMA enhancers are summarized in Table 1 below.

### 3.1. Retinoic Acid Derivatives

Retinoic acid, a vitamin A1 derivative, can mediate the growth and development function. Retinoic acid receptors (RARs) are identified as nuclear receptors. There are three isoforms of RARs in mammals, which are RARα, RARβ, and RARγ [98]. RARα is the most commonly expressed. RARs operate as transcriptional activators and repressors of many genes, influencing cellular processes such as differentiation, proliferation, and cellular homeostasis, all of which have been linked to CMA [99]. Previous research showed that the retinoic acid could cell-type-dependently stimulate macroautophagy by upregulating Beclin-1 and inhibiting the mTOR pathway. Anguiano J. et al.’s study explored the mechanism of retinoic acid on CMA. They identified the RARα receptor as a new CMA chemical target. The disruption of RARα signaling can stimulate the CMA process and inhibit macroautophagy [84]. All-trans retinoic acid (ATRA) is an activator of retinoic acid. The ATRA can mediate CMA through RARα signaling without affecting macroautophagy. The ATRA provides an opportunity to design RARα antagonists targeting CMA rather than autophagy. Atypical retinoid 7 (AR7), guanidine retinoid 1 (GR1), and guanidine retinoid 2 (GR2) are three CMA enhancers identified from the chemical designed retinoic acid derivatives library. AR7 and GR2 are the most effective CMA inducers by upregulating the LAMP2A level on the lysosomes [84]. Moreover, in LRRK2 knock-in primary neurons, AR7 can overcome the suppression effect of mutant LRRK2 on CMA-mediated α-synuclein clearance [85]. QX77 is a CMA activator derived from the AR7. It can mediate CMA by increasing the LAMP2A and Rab11 levels and further rescuing the lysosomal localization of LAMP2A at the lysosomal membrane of cystinotic *Ctns*-knock-out mouse embryonic fibroblasts and *CTNS*-knock-out human proximal tubule cells [86]. Bourdenx M. et al. modified the chemical structure of AR7 to make it suitable for in vivo administration and named the new compound CA77.1. CA77.1 can activate CMA in the mouse brain in the AD mouse model. Moreover, the Tau aggregates and amyloid plaques were decreased after the CA77.1 treatment [74]. 

### 3.2. Metformin

Metformin is the first line of pharmacotherapy for type 2 diabetes. Several studies have presented evidence suggesting that metformin may have some potential role other than glucose-lowering, such as antitumor, antiaging, cardiovascular protection, neuroprotection, or treatment for polycystic ovary syndrome [100]. Metformin has been identified as a CMA enhancer through drug screening approaches. Metformin activates CMA by upregulating lysosomes, HSC70, and LAMP2A, inducing the interaction between CMA substrates with HSC70 and LAMP2A [89]. Mechanistically, metformin activates transforming growth factor beta-activated kinase 1 (TAK1), which plays an important role in neuronal apoptosis and ND [101]. TAK1 is an IKKα/β kinases enhancer by phosphorylation at their Ser176/180 residues. Afterward, IKKα and IKKβ can phosphorylate HSC70 at Ser85. Thus, this study confirmed that metformin could activate CMA through TAK1-IKKα/β-HSC70 signaling [89]. By activating CMA, metformin alleviated the AD mice model’s brain Aβ load and behavioral abnormality. 

### 3.3. 6-Aminonicotinamide (6-AN)

6-AN is derived from a 6-aminonicotinic acid, and is an inhibitor of the NADP(+)-dependent enzyme, 6-phosphogluconate dehydrogenase. 6-AN suppresses glycolysis, induces ATP depletion, and works with DNA-crosslinking chemotherapeutic medicines such as cisplatin to destroy cancer cells [102]. A previous screening study tested total lysosomal protein breakdown rates on macroautophagy inhibitors, microtubule inhibitors, macroautophagy activators, protein synthesis inhibitors, glucose-6-phosphate dehydrogenase (G6PDH) inhibitor, and HSP90 inhibitor, suggesting the G6PDH inhibitor, 6-AN, and HSP90 inhibitor geldanamycin can activate CMA [90]. G6PDH is the pentose phosphate pathway enzyme. 6-AN treatment can reduce the intracellular level of NADPH produced by the pentose phosphate pathway. Based on the NADPH capacity to prevent oxidative damage and promote an oxidative environment, 6-AN can upregulate the level of oxidized cytosolic proteins and result in CMA activation [90]. On the other hand, G6PDH is one of the CMA substrates and regulates carbohydrate metabolism. Some results suggested that the dysregulation of carbohydrate metabolism is a potential mechanism in sporadic PD [103,104]. Moreover, an increased NAPDH level can be observed in the AD and PD brains [68]. Taken together, 6-AN-induced CMA rescues AD and PD phenotypes by regulating the oxidative environment and carbohydrate metabolism [68,90].

### 3.4. Geldanamycin (GA)

GA, an HSP90 inhibitor, was also identified as a CMA enhancer in Finn et al.’s screening [90]. GA binds to the ATP/ADP-binding pocket of HSP 90, impairing its function. In CMA, GA promotes the unfolded substrate proteins’ transport into the lysosome and increases the LAMP2A level and HSC70 level around the cell nucleus [90]. In addition, GA dramatically reduced mutant huntingtin aggregation in a cultured cell model of HD by inducing the HSP70 and HSP40 heat shock responses [105]. GA can activate CMA and result in downregulation of the steady-state levels of ryanodine receptor 2 (RyR2) [106]. In ND, loss of RyR2 in hippocampal pyramidal neurons affects memory learning and the activity-evoked structural plasticity of dendritic spines [107].

### 3.5. Bortezomib

Bortezomib is a boronic acid dipeptide derivative and a proteasome inhibitor, the first proteasome inhibitor to be used to treat cancer. Bortezomib is typically used to treat multiple myeloma (MM) and mantle cell lymphoma [108]. In a bortezomib resistance study, bortezomib confirmed the upregulation capacity in CMA. They found that bortezomib-induced endoplasmic reticulum stress is the main factor of CMA activation by evaluating the LAMP2A protein level [109]. Although bortezomib treatment benefits MM, the bortezomib-induced peripheral neuropathy (BiPN) in MM patients is a novel issue. In another study, some candidate drugs were combined and treated with bortezomib to reduce BiPN [91]. They discovered that CMA could be a potential mechanism of aggregation clearance in Schwann cells in BiPN. The combined bortezomib treatment with suberoylanilide hydroxamic acid (SAHA), 17-allylamino-17-demethoxy-geldanamycin (17-AAG), or clonazepam (CZP) indued colocalization of LAMP2A, HSC70, and aggregated proteins in a rounded structure. In a phase I trial of the bortezomib combined treatment with SAHA for MM patients, the PN symptom was rarely reported [91]. PN has been identified in PD with the phosphorylated α-synuclein accumulation feature [110]. The aggregation clearance capacity of the bortezomib combined treatment can potentially alleviate the burden of disease exerted by PN on PD. 

### 3.6. Manganese (Mn)

Mn, a crucial element for humans, animals, and plants, is essential for human health, as it is required for growth, metabolism, and the antioxidant system [111]. Nevertheless, elevated levels of the metal can affect neurotoxicity and further result in ND [112]. Yan et al.’s research ran an experiment on the role of α-synuclein in Mn-mediated neurocyte injury in wild-type and *Snca*-knock-out mice. Compared with wild-type mice, the *Snca*-knock-out mice were more susceptible to Mn toxicity. These results suggest a neuroprotective role of α-synuclein against Mn-related neurotoxicity. As a CMA substrate, α-synuclein provides a possible link between Mn and CMA. Low-dose Mn can upregulate the LAMP2A and HSC70 levels and activate the CMA process [92]. In addition, Mn treatment can enhance the interaction of LAMP2A and α-synuclein, which suggests that degradation of Mn-mediated α-synuclein overexpression depends on the CMA pathway [92].

### 3.7. Trehalose

Trehalose is a nonreducing disaccharide with two glucose units connected by an α,α-1,1-glycosidic bond. Trehalose is considered a neuroprotective reagent in some ND animal models such as PD and HD. Several studies suggested that trehalose is an autophagy inducer that can reduce toxic protein aggregates in ND [113]. Previous research has shown that the transcription factor EB (TFEB) mediated trehalose-induced autophagy. This result showed that trehalose could affect the expression of Bcl-2-associated athanogene 3 (BAG3), a CMA protein [93]. In another study, trehalose reverted the epoxomicin-induced damage in the HD model. Based on the previous results, they ran an experiment to detect the effects of trehalose in macroautophagy and CMA. Thus, the activation role of trehalose in CMA was confirmed by testing the HSC70 and LAMP2A levels. Trehalose increases CMA activation in normal conditions and HD models [114].

### 3.8. Caffeine

Caffeine is a xanthine alkaloid compound. It is a CNS stimulant drug found in tea, coffee, and cocoa [115]. Generally, caffeine reduces the PD risk in epidemiological studies and reports. Luan et al. found that chronic caffeine can reduce the α-synuclein-induced pathological changes in the A53T α-synuclein fibril PD model. In addition to the reduction in α-synuclein, caffeine also has the capacity to regulate cellular apoptosis and activation of microglial and astrocytic in the striatum [94]. Afterward, they tried to determine the mechanism of protein aggregate removal after chronic caffeine treatment. At first, the UPS activity was examined by the PSMC3 and PSMB6 protein levels. This result demonstrates that chronic caffeine has no impact on UPS. Then, the effect of caffeine on the autophagy-lysosome pathway was examined. The results indicate that A53T α-synuclein fibrils caused abnormalities in macroautophagy and CMA. Chronic caffeine preferentially reversed α-synuclein-induced defects in macroautophagy by increasing LC3-II and decreasing SQSTM1/p62. The most important is that caffeine selectively reverses the defects of CMA. Chronic caffeine rescued the α-synuclein-induced LAMP2A decrease. The evidence also provides another potential target, which is the adenosine receptors. Adenosine receptors and other pharmacological targets may induce caffeine regulation of autophagy [94].

### 3.9. β-Asarone

β-asarone, an extraction from *Acorus Tatarinowii* Schott, has a protective effect on ND. β-asarone is related to several autophagic regulators such as JNK, p-JNK, Bcl-2, Beclin-1, and α-synuclein [116]. Research showed that β-asarone could downregulate the α-synuclein level and upregulate the MEF2D level in the 6-OHDA-induced PD model. As it is known that MEF2D-HSC70 binding has been identified as the primary regulator for CMA, they further examined the level of HSC70, LAMP2A, and MEF2D in the mesencephalon of the 6-OHDA-induced PD rats. The results showed that in addition to the MEF2D level, HSC70 and LAMP2A were increased in this PD model, indicating that β-asarone is a CMA enhancer [95]. This study also identified the HSP70-MAPK-MEF2D-Beclin-1 signaling pathway as the β-asarone treatment pathway [95].

### 3.10. Silymarin

Silymarin is the active component of *Silybum marianum*, an ancient medicinal herb with hepatoprotection and antioxidant features. Silymarin is a potential small molecule for the treatment of AD. Previous research indicated that silymarin has antioxidant properties in the CNS, which allow it to reach the CNS via the blood-brain barrier [117]. In the MPTP mouse model, silymarin could rescue the antioxidant defense system in dopamine-producing neurons of the nigrostriatal area. Based on this finding, Tripathi et al. ran a series of research on autophagy-related proteins. The results demonstrated that silymarin could inhibit MPTP-induced proteins such as Beclin-1, sequestosome, p-AMPK, p-ULK1, and α-synuclein. Moreover, silymarin can also upregulate LAMP2, p-mTOR, LAMP2A, and dopamine [96]. These results indicate that silymarin can prevent oxidative stress and mitochondrial dysfunction through autophagy. CMA is activated in excessive oxidative stress conditions. Silymarin increased the LAMP2A level and reduced α-synuclein in the MPTP mouse [96]. Overall, silymarin can enhance functional CMA activity and induce a neuroprotection role.

### 3.11. Dihydromyricetin (DHM) and Salvianolic acid B (Sal B)

DHM is a bioflavonoid derived from *Ampelopsis grossedentata.* They have a variety of bioactive properties such as antioxidant, antibacterial, antiviral, anti-inflammatory, anti-cancer, and neuroprotective properties [118]. DHM is identified as a neuroprotective drug as it can inhibit the α-synuclein fibrillogenesis and induce autophagy [119,120]. Sal B, a compound extracted from *Salvia miltiorrhiza* (*Danshen*), is widely known for its anti-oxidative properties [121]. Sal B has recently been linked to a reduction in amyloid protein fibril formation and decrease in neuroinflammation, which suggests a neurological function in mouse models of ND [122]. In Wu et al.’s research, they found that DHM and Sal B affect α-synuclein aggregation. To detect the role of CMA-mediated destruction of aggregated α-synuclein and the possible function of autophagy controlled by DHM and Sal B, they tested the level of WT α-synuclein, SynT, LAMP1, and LAMP2A after DHM and Sal B treatment both in in vitro and in vivo models. The increased levels of LC3-II and LAMP2A suggested that DHM and Sal B can activate CMA and macroautophagy. DHM- and Sal B-activated CMA can degrade α-synuclein aggregation and the toxicity of α-synuclein aggregation. In addition, they also found inhibition of astrogliosis and microgliosis in vivo by DHM and Sal B treatment [97]. Taken together, DHM and Sal B have the capacity to activate CMA and regulate α-synuclein aggregation, further demonstrating the therapeutic potential of DHM and Sal B in PD.

### 3.12. Mycophenolic Acid (MPA)

MPA, produced by the *Penicillium* fungus, is one of the IMPDH inhibitors and a regulator of ATP and GTP synthesis [123]. Different from the other small molecules, MPA does not target any CMA substrates. It activates CMA by selectively inhibiting GTP synthesis [16]. GFAP and EF1α act as regulatory proteins in CMA processing. The EF1α-GTP binding can mediate the release of EF1α. Bandyopadhyay et al. demonstrated that MPA could deplete GTP. As a feedback of GTP reduction, the release of lysosome-associated EF1α and self-association of GFAP are blocked to inhibit LAMP2A multimerization disassembly, thereby enhancing CMA activity [16]. 

## 4. Conclusions

As a type of selective autophagy, the molecular mechanism of CMA has been intensively studied, and its implication in ND is being explored. CMA can be observed in NDs such as PD, AD, HD, ALS, FTLD, and prion disease. However, the molecular mechanism of CMA impairment and its dynamic changes in ND are still poorly understood and deserve further investigation. Specifically, what are the changes in the upstream regulators of HSC70 and LAMP2A transcription in the ND? Are post-translational modification and the interacting partners of CMA regulators altered in the ND? Is there any brain region and cell type difference in CMA activity in the brain of ND? Activation of the CMA process in NDs has emerged as a potential therapeutic strategy. So far, several compounds have been reported as enhancers of CMA and tested in ND models. However, most compounds have limited selectivity on CMA, and the molecular mechanism is rarely dissected. The identification of highly selective CMA enhancers with clear drug targets is crucial in pharmacologically modulating CMA for ND therapy.

## Figures and Tables

**Table 1:** Chaperone-mediated autophagy (CMA)-inducing small molecules and their pharmacological effects in neurodegenerative disease (ND).

Small Molecule	CMA Regulation Mechanism *	ND Models	Pharmacological Effects	Refs.
AR7 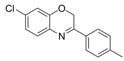	LAMP2A ↑ LRRK2 ↓	Mouse fibroblasts overexpressing α-synuclein Primary cortical neurons (DIV9) from LRRK2^R1441G^ knock-in mutant mice	Reduced α-synuclein	[84,85]
QX77 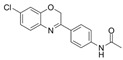	LAMP2A ↑ Rab11 ↑	BV2 cells; Primary microglia; PD astrocytes	Restored the degradation of α-synuclein	[86,87,88]
CA77.1 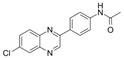	LAMP2A ↑	NIH 3T3 cells expressing the KFERQ-PS-Dendra reporter; PS19 transgenic mice; Tg mice	Reduced β-amyloid and Tau pathologies and glial activation.	[74]
Metformin 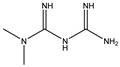	HSC70 ↑	PC12 cells incubated with Aβ; APP/PS1 mouse model	Reduced cytotoxicity of APP and Aβ	[89]
6-aminonicotinamide 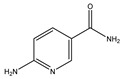	NADPH ↓	IMR-90	Decreased cellular NADPH levels	[90]
Geldanamycin 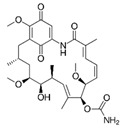	LAMP2A ↑ HSC70 ↑	IMR-90; Fibroblasts; COS-1 cells expressing EGFR and huntingtin exon 1 protein with 72 glutamines	Stimulated RyR2 degradation Inhibited fibrillar protein aggregation	[90]
Bortezomib combination treatment with SAHA, 17-AAG, or CZP 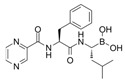	LAMP2A ↑ HSC70 ↑	Schwann cells	Aggregation clearance	[91]
Manganese Mn	LAMP2A ↑ HSC70 ↑	Wild-type and *Snca* knock-out mice	Reduced α-synuclein	[92]
Trehalose 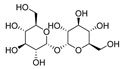	LAMP2A ↑ HSC70 ↑	HD fibroblasts	Reduced protein accumulation	[93]
Caffeine 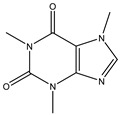	LAMP2A ↑	A53T α-synuclein fibril model of PD	Reduced α-synuclein	[94]
β-asarone 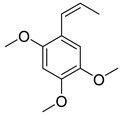	MEF2D ↑	6-OHDA-induced PD rats	Reduced α-synuclein	[95]
Silymarin 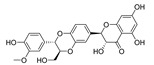	LAMP2A ↑	MPTP mouse model	Reduced α-synuclein	[96]
Dihydromyricetin 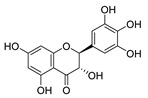	LAMP2A ↑	H4 cell model expressing SynT-aggregation; Bacterial Artificial Chromosome (BAC) transgenic mice	Reduced α-synuclein	[97]
Salvianolic acid B 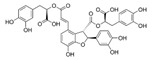	LAMP2A ↑	H4 cell model expressing SynT-aggregation; Bacterial Artificial Chromosome transgenic mice	Reduced α-synuclein	[97]
Mycophenolic acid 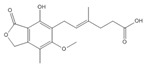	GTP ↓	RALA cells	Reduce release of EF1α and self-association of GFAP	[16]

* ↑—increase; ↓—decrease.

## Data Availability

Not applicable.
